# Teaching Telepsychiatry Skills: Building on the Lessons of the COVID-19 Pandemic to Enhance Mental Health Care in the Future

**DOI:** 10.2196/37939

**Published:** 2022-10-14

**Authors:** Katharine Smith, John Torous, Andrea Cipriani

**Affiliations:** 1 Department of Psychiatry University of Oxford Oxford United Kingdom; 2 Oxford Health NHS Foundation Trust Warneford Hospital Oxford United Kingdom; 3 Oxford Precision Psychiatry Lab Oxford Health Biomedical Research Centre Oxford United Kingdom; 4 Division of Digital Psychiatry Beth Israel Deaconess Medical Center Harvard Medical School Boston, MA United States

**Keywords:** mHealth, mental health, smartphones, telehealth, telepsychiatry, COVID-19

## Abstract

COVID-19 has accelerated the use of telehealth and technology in mental health care, creating new avenues to increase both access to and quality of care. As video visits and synchronous telehealth become more routine, the field is now on the verge of embracing asynchronous telehealth, with the potential to radically transform mental health. However, sustaining the use of basic synchronous telehealth, let alone embracing asynchronous telehealth, requires new and immediate effort. Programs to increase digital literacy and competencies among both clinicians and patients are now critical to ensure all parties have the knowledge, confidence, and ability to equitably benefit from emerging innovations. This editorial outlines the immediate potential as well as concrete steps toward realizing the potential of a new, more personalized, scalable mental health system.

During the COVID-19 pandemic, telepsychiatry—a “virtually perfect” solution to the immediate crisis of a global pandemic [1]—has provided an effective way to deliver care while maintaining social (or, more accurately, physical) distancing. Although remote assessments were novel to many real-world clinicians, telepsychiatry is not a new discipline. There is a well-established evidence base of effectiveness in different patient populations and demographics [2]. Established guidance on telepsychiatry is available and has been synthesized into a user-friendly format, updated to include COVID-19–specific strategies [3,4]. There are additional advantages over in-person treatment in terms of convenience, privacy, reduced stigma, and ease of integration with multidisciplinary viewpoints and specialized care, as well as with other digital technologies [5-7]. Feedback from patients is also positive [8,9] and a majority want to continue to use it after the pandemic [10]—but can the same be said for clinicians? To use fully the wide range of modalities for treatment delivery including telepsychiatry and digital approaches, and to feel confident and competent in offering a truly hybrid service, clinicians will need training to build on the immediate experience they gained during the COVID-19 pandemic [9,11].

At the beginning of the pandemic, there was an almost overnight transition to video- and telephone-based assessments in mental health in many countries [12,13]. Immediate challenges were related to technology and access issues. These included access to both sufficient broadband speed and to a software solution compliant with local and national guidance, which varies across regions and countries. For example, in the United States, software must be compliant with the Health Insurance Portability and Accountability Act (HIPAA) [14], whereas in other countries such as the United Kingdom, commonly available tools such as Skype, WhatsApp, and FaceTime are deemed acceptable, assuming an appropriate local risk assessment. Audio and video transmission also need to be encrypted (according to national guidance) and the device used needs security features (such as passphrases, two-factor authentication, and the latest antivirus, antimalware, and firewall software with updates) [5]. Licensing and legislation also initially provided a barrier, especially in the United States, as prior to the COVID-19 pandemic, physicians in telemedicine were required to be licensed in the state in which the patient was located. In the context of the pandemic, these barriers were quickly overcome (eg, by emergency waivers) to allow telepsychiatry to proceed. Clinicians quickly modified their in-person skills to using telephone and video, learning to a large extent by “doing” [15]. Still, not all were able to adapt easily, and a lack of digital competency has been suggested to be a major source of burnout and stress in these clinicians [16].

However, there is much more to telepsychiatry than just video visits and phone calls. Video and telephone approaches are often classed as “synchronous” telepsychiatry because the interaction, although distanced, is delivered in real time. Asynchronous telepsychiatry, by contrast, occurs when the clinician and patient interaction is separated by time as well as distance. Examples include use of apps for monitoring or delivering treatment, and use of smartphones and other mobile health apps [17]. These technologies can all add rich data and modes of communication to the clinical consultation, but clinicians need to be aware of potential pitfalls as well as advantages. During the pandemic, clinicians have focused almost invariably on video and telephone consultations only [13]. Even after more than two years of pandemic restrictions, clinicians continue to have less experience around asynchronous telehealth, despite its potential to exponentially increase access to care.

Going forward, it is clear that telepsychiatry has the potential to offer much more than a simple replacement of face-to-face care, as a short-term solution to an immediate crisis. Telepsychiatry can now be used toward its true potential in radically increasing access to care as well as quality through an artful combination of synchronous and asynchronous technologies [18], but to realize this, the art of telepsychiatry requires investments in teaching the knowledge, skills, and competencies necessary to use the full range of these technologies. This extension of skills is key, as telepsychiatry will be needed more and more in the future, not only as an essential element of planning for the next crisis but also as the most efficient and effective approach to move psychiatry toward personalized and preventive care that serves the entire population.

Retraining the workforce need not start from scratch. Synchronous telepsychiatry [19], mobile technologies and apps [17,20], social media [21,22], and digital informatics [23] already have proposed competency frameworks. Many are aligned with pre-existing medical education frameworks (see Multimedia Appendix 1 for examples) and use levels of skill attainment: Novice, Competent/Proficient, and Expert. These proposed competencies focus on acquiring and developing skills rather than pure knowledge acquisition [24], and skills development is monitored through ongoing assessment during patient care [25]. Telehealth curricula have been proposed for medical students and for residents (Multimedia Appendix 1). There are examples of programs for teaching telehealth [26-37] (Multimedia Appendix 2), and some psychiatry residency programs in the United States for example are also offering informatics tracks [38]. Novel approaches, such as identifying a care team member (a “digital navigator”) to promote and model digital health within clinical teams [39] also show great promise. However, there are important challenges with training the current workforce in telepsychiatry:

1. Enthusiasm may vary and individual clinicians differ in their openness to training. Many already feel they are competent enough, given their experience during the COVID-19 pandemic. The focus of their immediate management has used synchronous techniques, and clinicians may assume that skills can be translated directly from the in-person to virtual setting. Digital literacy among clinicians can also vary [40] and depends to some extent on age [41]. Younger staff and students are often considered as “digital natives” who have grown up with widespread digital technologies, compared to so-called “digital immigrants” who did not encounter these until adulthood [12,21]. However, these are broad categories, with individual variation in skills and enthusiasm for digital literacy, and significant differences for people in their use of and comfort with technologies. Teaching must be targeted and tailored, taking these baseline differences into account.
2. Clinicians are only part of the equation; we also need to improve access to technology for patients. A successful virtual or hybrid clinical interaction can only occur if both clinician and patient are able to access and navigate the tools being used. Access to devices and adequate connectivity represents one significant digital divide, but there is also a second barrier, in which patients have access to technologies but do not have the digital literacy, competence, or confidence to use them to their full potential. Offering training and skills building to patients to strengthen their competence and autonomy in using digital technology to support their health and their therapeutic relationship is critical to ensure access to help for those who need it the most [18]. There are successful examples of schemes for those with serious mental illness, with skills and competencies that are shareable for other groups to modify or expand on as needed [42], and those for community stakeholders to enable remote participation in research studies or community engagement initiatives [43].
3. Existing competency frameworks are comprehensive and detailed, but many have not actually been implemented. This makes them ideally suited for further testing, but less appropriate for broad implementation today. These competencies that have been outlined already could be adapted and amalgamated into a pragmatic time-limited approach that is acceptable to all levels of pre-existing skill and interest [19].
4. Synchronous and asynchronous telepsychiatry have many similarities. For example, their common goal is to enable a clear and therapeutic exchange of information, while preserving professional boundaries [20]. However, there are also a number of key differences, which may require slightly different skill sets. This is partly because asynchronous interactions are often spontaneous and unstructured, and may occur outside health settings and their associated platforms such as the electronic health record. This generates more potential boundary, legal, and ethical issues, which clinicians will need to actively manage. Clinicians and patients need to be aware of both the advantages and potential pitfalls of these different modes of communication (eg, email, portals, messaging), and successfully navigate any potential overlap between personal and professional life. Social media is another aspect where specific training is critical, especially as younger patients are likely to be influenced by it. Increased digitalization has expanded the boundaries and tools available in medical practice, but specific training has not kept up with increased use of technology. Potential hazards include breaches of confidentiality, privacy, and professional boundaries [44], but these need to be balanced against the potential benefits, such as increased opportunities for professional networking, collaboration, and education and training, and increased patient engagement, education, and health promotion [21,44]. Organizations have developed professional guidelines, standards, and consensus statements regarding responsible physician use of social media and the internet (see Multimedia Appendix 3 for examples), but given the wide variety of guidance documents, in practice it has been difficult for clinicians to absorb the knowledge, competence, and skills required to use social media, apps, and wearables in clinical interactions [20,22]. Novice and more advanced learners alike require competency-based education in this area. Experienced clinicians may be less confident than trainees in digital literacy and may require more training in the benefits and potential pitfalls of different digital media [21], but all aspects of skills will need to be addressed during training to achieve a level of competence to safely blend a range of techniques.

To meet these challenges, training will need to be evidence-based, relevant for the challenges of a post–COVID-19 world, and also engaging for clinicians. Competencies will need to measurable to assess change and there will need to be ongoing evaluation, including feedback from patients. Appetite and interest will vary, and practicing clinicians will already have gained sufficient experience during the pandemic to have progressed beyond the “Novice” stage; therefore, a two-level process aiming for the competency levels of “Competent/Proficient” and “Expert” (for those who wish) would be a helpful model. There is no doubt that developing a telepsychiatry teaching program is warranted, but it will be a challenging process. However, much of the hard work has been completed in developing guidance and a range of competencies. The pandemic has accelerated telepsychiatry into a commonly used, effective, and acceptable route for mental health consultation. Now is the time to complete the translational pathway and allocate dedicated research funding. We need to grasp this impetus and extend skills and competencies into the full range available, so we can offer the very best combination of approaches and treatments to our patients.

## Appendix

Multimedia Appendix 1 Examples of medical education frameworks.

Multimedia Appendix 2 Examples of teaching telehealth.

Multimedia Appendix 3 Examples of guidelines for professionalism, social media, and the internet.
