# Supplementary material for: Teaching Telepsychiatry Skills: Building on the Lessons of the COVID-19 Pandemic to Enhance Mental Health Care in the Future
Source: JMIR Ment Health. 2022 Oct 14;9(10):e37939. doi: 10.2196/37939 (PMC9617186; doi:10.2196/37939)
Supplement: Multimedia Appendix 1 [file mental_v9i10e37939_app1.docx]

**Multimedia Appendix 1.** Examples of medical education frameworks.

| Name | Competencies/domains | Website |
| --- | --- | --- |
| Accreditation Council for Graduate Medical Education (ACGME) | Patient care, medical knowledge, practice-based learning and improvement, systems-based practice, professionalism, and interpersonal skills and communication | [Common Program Requirements (acgme.org)](https://www.acgme.org/What-We-Do/Accreditation/Common-Program-Requirements/) |
| The evidence-based CanMEDS framework | Describes the knowledge, skills, and abilities that specialist physicians need for better patient outcomes, based on the seven roles that all physicians play: medical expert, communicator, collaborator, manager, health advocate, scholar, and professional. | [CanMEDS Framework: The Royal College of Physicians and Surgeons of Canada](https://www.royalcollege.ca/rcsite/canmeds/canmeds-framework-e) |
| Association of American Medical Colleges (AAMC). | Medical knowledge, patient care skills, and attitudes, interpersonal and communication skills and attitudes, ethical judgment, professionalism, lifelong learning and experience-based improvement, and community and systems-based practice. | [Tomorrow's Doctors, Tomorrow's Cures \| AAMC](https://www.aamc.org/)  [Toward a Common Taxonomy of Competency Domains for the Healt... : Academic Medicine (lww.com)](https://journals.lww.com/academicmedicine/Fulltext/2013/08000/Toward_a_Common_Taxonomy_of_Competency_Domains_for.21.aspx) |
| AAMC: Telehealth Competencies Across the Learning Continuum | - Patient Safety and Appropriate Use of Telehealth - Access and Equity in Telehealth - Communication via Telehealth - Data Collection and Assessment via Telehealth - Technology for Telehealth - Ethical Practices and Legal Requirements for Telehealth.   Three tiers that represent developmental stages in physician development: 1) entry to residency or recent medical school graduate, 2) entry to practice or recent residency graduate, and 3) experienced faculty physician or three to five years post-residency. | [New and Emerging Areas in Medicine Series Telehealth Competencies Across the Learning Continuum (aamc.org)](https://store.aamc.org/downloadable/download/sample/sample_id/412/) |
