# Supplementary material for: Teaching Telepsychiatry Skills: Building on the Lessons of the COVID-19 Pandemic to Enhance Mental Health Care in the Future
Source: JMIR Ment Health. 2022 Oct 14;9(10):e37939. doi: 10.2196/37939 (PMC9617186; doi:10.2196/37939)
Supplement: Multimedia Appendix 2 [file mental_v9i10e37939_app2.docx]

**Multimedia Appendix 2.** Examples of teaching telehealth.

| Country  [reference] | Methods | Results |
| --- | --- | --- |
| **Medical students** | | |
| United States [26] | Mixed methods review – literature review and email approach. | - >25% of medical schools have preclinical telemedicine training - Almost 50% incorporate telemedicine exposure into students’ clerkship curriculum - Implementation of teaching varies between institutions. |
| Australia, Brazil, United Kingdom, United States, and Japan/Pacific Islands [27] | Literature review of providing telehealth training | - 388 papers identified, 9 studies selected for final review. - Education and training in telehealth has been provided at university level and as vocational courses - Reported curriculum items included terminology, clinical applications, the evidence-base, and technological aspects. |
| United States [28] | Strategies described for effectively integrating medical students into telehealth visits |  |
| United States [29]  Third-year medical students (n=149) | - asynchronous lectures covering telehealth history, applications, ethics, safety, military uses, etiquette, and patient considerations - in-person interactive telehealth instruction including patient selection, current uses, and risk management - supervised mock patient telehealth encounters - hands-on diagnosis and advanced surgical procedures using telehealth equipment | 10.1% average improvement in:   - knowledge between pre and post-test scores - completion of competency-based checklists - post-course preceptor and student feedback   80% indicated future plans to practice telehealth |
| Australia [30]  Third-year medical students | Training in:   - methods of conducting teleconsultations - legal and ethical considerations - technical and procedural issues - barriers and benefits for patients and doctors. | Self-reported improvements in understanding and procedures, and in confidence in conducting a telehealth consultation. |
| Germany [31]  Third-year medical students | 3-week curricular module on digital health | Overall positive feedback from students and lecturers |
| United States [32]  Third-year medical students | - complete the American College of Physicians (ACP) module on telehealth - orientation via the Zoom online platform by clinical faculty - weekly telehealth clinic schedule, precepted by residents and attendings in Internal Medicine. | - Baseline survey: 90% of students believed the telemedicine experience would be a valuable addition. - Most were confident that, with training, they could effectively complete a telemedicine visit. - 80% felt that telehealth would play an important role in their future careers. |
| United States [33]  Preclinical and clinical students  Focus on social media | - survey of medical students regarding SM use, rationale for and frequency of use, and concerns - a workshop-format piloted for preclinical students on online professionalism - longitudinal social media–based curriculum piloted for clinical students |  |
| **Residents/doctors in training** | | |
| Canada [34]  Psychiatry residents | Qualitative interview-based study into areas of importance for telepsychiatry training in postgraduate psychiatry residency. 16 interviews of faculty and residents, to reflect on their experiences. Data thematically analyzed. | Telepsychiatry skills identified through this study map onto the CanMEDS roles, particularly the roles of medical expert, communicator, collaborator, leader and health advocate. |
| United States [35]  Psychiatry residents | 3 video vignettes, created as an interactive teaching tool to augment the AADPRT Professionalism and the Internet curriculum.  Aimed for trainees (to gain a better understanding of e-professionalism breaches), and provide tools for senior clinicians to address them when they arise in training | Plan to implement these as a pilot program, then to develop a video vignette-based curriculum to facilitate teaching e-professionalism in training. |
| United States [36]  Neurology Residents (third and fourth years) | Pilot of an experiential outpatient teleneurology curriculum. Interactive lecture and 4 weeks of teleneurology clinics. | Residents' performance on knowledge quizzes improved from 53% to 88%. Confidence also improved. All felt more competent using telemedicine for patient care in their eventual career. |
| United States [37]  Internal medicine Residents | - introduction to telemedicine equipment in the first year - didactic learning in the second year - experiential learning through remote monitoring of chronic disease in the third year. | Improved self-reported knowledge and quality of care.  Improved self-reported communication, comfort and perceived ability to provide telehealth services after participation  41% of Residents felt their ability to utilize telehealth as part of their current or future practice was greater than average after completion compared to 2% at baseline. |
