# Supplementary material for: Teaching Telepsychiatry Skills: Building on the Lessons of the COVID-19 Pandemic to Enhance Mental Health Care in the Future
Source: JMIR Ment Health. 2022 Oct 14;9(10):e37939. doi: 10.2196/37939 (PMC9617186; doi:10.2196/37939)
Supplement: Multimedia Appendix 3 [file mental_v9i10e37939_app3.docx]

**Multimedia Appendix 3.** Examples of guidelines for professionalism, social media and the internet.

| Institution | Curriculum | Reference URL |
| --- | --- | --- |
| American Association of Directors of Psychiatry Residency Training | Curriculum on professionalism and the Internet | <https://pubmed.ncbi.nlm.nih.gov/29949054/> |
| American College of Physicians | Online Medical Professionalism: Patient and Public Relationships: Policy Statement from the American College of Physicians and the Federation of State Medical Boards | <https://pubmed.ncbi.nlm.nih.gov/23579867/> |
| American Medical Association | Guidelines for Patient-Physician Electronic Mail and Text Messaging | <https://policysearch.ama-assn.org/policyfinder/detail/Guidelines%20for%20Patient-Physician%20Electronic%20Mail%20and%20Text%20Messaging%20H-478.997?uri=%2FAMADoc%2FHOD.xml-0-4344.xml> |
| American Medical Association | Professionalism in the use of social media. | <https://www.ama-assn.org/delivering-care/ethics/professionalism-use-social-media> |
| American Psychiatric Association | The Internet in clinical psychiatry (resource document), Joint Reference Committee | <https://www.psychiatry.org/File%20Library/Psychiatrists/Directories/Library-and-Archive/resource_documents/Resource-2014-Telepsychiatry-Clinical-Psychiatry.pdf> |
| Australian Medical Association | Social media and the medical professionalism | <https://www.ama.com.au/sites/default/files/2021-04/2020%20AMA%20Social%20Media%20Guide.pdf> |
| British Medical Association | Social media use: Practice and ethical guidance for doctors and medical students | <https://www.bma.org.uk/advice/employment/ethics/social-media-guidance-for-doctors>  <https://www.bma.org.uk/advice/employment/ethics/medical-students-ethics-toolkit/12-students-and-social-media> |
| Council of Residency Directors Social Media Task Force | Social media guidelines and best practices | <https://pubmed.ncbi.nlm.nih.gov/24578765/> |
| Federation of State Medical Boards, Inc. | Model policy guidelines for the appropriate use of social media and social networking in medical practice | <https://www.policymed.com/2012/06/federation-of-state-medical-boards-model-policy-guidelines-for-social-media.html> |
| General medical Council (UK) | Guidance: Doctors' use of social media | <https://www.gmc-uk.org/ethical-guidance/ethical-guidance-for-doctors/doctors-use-of-social-media/doctors-use-of-social-media> |
